# Mutations in IFT-A satellite core component genes *IFT43* and *IFT121* produce short rib polydactyly syndrome with distinctive campomelia

**DOI:** 10.1186/s13630-017-0051-y

**Published:** 2017-04-10

**Authors:** Ivan Duran, S. Paige Taylor, Wenjuan Zhang, Jorge Martin, Faisal Qureshi, Suzanne M. Jacques, Robert Wallerstein, Ralph S. Lachman, Deborah A. Nickerson, Michael Bamshad, Daniel H. Cohn, Deborah Krakow

**Affiliations:** 1 Department of Orthopaedic Surgery, David Geffen School of Medicine at the University of California at Los Angeles, Los Angeles, CA 90095 USA; 2 Department of Human Genetics, David Geffen School of Medicine at the University of California at Los Angeles, Los Angeles, CA 90095 USA; 3 Department of Obstetrics and Gynecology, David Geffen School of Medicine at the University of California at Los Angeles, Los Angeles, CA 90095 USA; 4 Department of Molecular, Cell, and Developmental Biology, University of California, Los Angeles, Los Angeles, CA 90095 USA; 5 International Skeletal Dysplasia Registry, University of California, Los Angeles, Los Angeles, CA 90095 USA; 6 Department of Pathology, Hutzel Women’s Hospital/Wayne State University, Detroit, MI 48201 USA; 7 Kapi’olani Medical Center for Women and Children, Honolulu, HI 96826 USA; 8 University of Washington Center for Mendelian Genomics, University of Washington, Seattle, WA 98195 USA; 9 Networking Research Center on Bioengineering, Biomaterials and Nanomedicine, (CIBER-BBN), University of Malaga, Málaga, Spain

**Keywords:** Short rib polydactyly syndrome, SRPS, Cilia, Skeletal ciliopathy, IFT43, IFT121, Intraflagellar transport, IFT, IFT-A complex, Retrograde transport, Cartilage

## Abstract

**Background:**

Skeletal ciliopathies comprise a spectrum of ciliary malfunction disorders that have a profound effect on the skeleton. Most common among these disorders is short rib polydactyly syndrome (SRPS), a recessively inherited perinatal lethal condition characterized by a long narrow chest, markedly shortened long bones, polydactyly and, often, multi-organ system involvement. SRPS shows extensive locus heterogeneity with mutations in genes encoding proteins that participate in cilia formation and/or function.

**Results:**

Herein we describe mutations in *IFT43*, a satellite member of the retrograde IFT-A complex, that produce a form of SRPS with unusual bending of the ribs and appendicular bones. These newly described *IFT43* mutations disrupted cilia formation, produced abnormalities in cartilage growth plate architecture thus contributing to altered endochondral ossification. We further show that the *IFT43* SRPS phenotype is similar to SRPS resulting from mutations in the gene encoding IFT121 (WDR35), a direct interactor with IFT43.

**Conclusions:**

This study defines a new *IFT43*-associated phenotype, identifying an additional locus for SRPS. The data demonstrate that IFT43 is essential for ciliogenesis and that the mutations disrupted the orderly proliferation and differentiation of growth plate chondrocytes, resulting in a severe effect on endochondral ossification and mineralization. Phenotypic similarities with SRPS cases resulting from mutations in the gene encoding the IFT43 direct interacting protein IFT121 suggests that similar mechanisms may be disrupted by defects in these two IFT-A satellite interactors.

**Electronic supplementary material:**

The online version of this article (doi:10.1186/s13630-017-0051-y) contains supplementary material, which is available to authorized users.

## Background

A spectrum of skeletal dysplasias results from mutations in genes that participate in primary cilia formation and/or function. These disorders are referred to as ciliary disorders of the skeleton or skeletal ciliopathies [1]. The disorders exhibit overlapping clinical and radiographic findings, but within this group there are distinct phenotypes that include short rib polydactyly syndrome (SRPS), asphyxiating thoracic dystrophy (ATD) or Jeune syndrome, Sensenbrenner syndrome or cranioectodermal dysplasia (CED), Ellis-van Creveld syndrome (EVC), and Weyers acrofacial dysostosis [1]. Among these, the perinatal lethal SRPS is the most severe and is characterized by a long narrow thorax, markedly shortened long bones and frequent polydactyly. Extra-skeletal defects also occur in the neurologic, cardiac, gastrointestinal, and genitourinary systems. SRPS radiographic findings include horizontal ribs, an abnormal acetabulum, small iliac bones, hypoplastic or absent fibulae and disharmonious ossification of the skeleton. Based primarily on radiographic findings, SRPS has been further subdivided into type I/III (Saldino-Noonan/Verma-Naumoff), type II (Mohr-Majewski), and type IV (Beemer-Langer) [1]. Asphyxiating thoracic dystrophy (ATD) is phenotypically similar to SRPS, but with less severe overall features, and about one-third of affected individuals survive the neonatal period [2].

Mutations in a variety of ciliary genes have been associated with SRPS/ATD phenotypic spectrum, reflecting the different components of ciliary function including those are involved in anterograde and retrograde intraflagellar transport, *IFT172* [OMIM 607386], *IFT80* [OMIM 611177], *IFT52* [OMIM 617094], *IFT81* [OMIM 605489], *IFT140* [OMIM 614620], IFT144/*WDR19* [OMIM 608151], *DYNC2H1* [OMIM 603297], *DYNC2LI1 [OMIM 617083]*, *WDR34* [OMIM 613363], *WDR60* [OMIM 615462], *TCTEX1D2*, *IFT121/WDR35* [OMIM 613602], *TTC21B* [OMIM 612014], and those that have centrosomal roles, *KIAA0586* [OMIM 610178], *CEP120* [OMIM 613446], *ICK* [OMIM 612325], *INTU* [OMIM 610621], and *NEK1* [OMIM 604588] [3–22]. Mutations in several of the same genes that produce SRPS and ATD also result in CED, a related nonlethal autosomal recessive disorder primarily distinguished from ATD by craniofacial and ectodermal abnormalities that include craniosynostosis, dolichocephaly, telecanthus, low set ears, hypo/microdontia, abnormal nails and hair, and a high incidence of development of renal symptoms [23]. Mutations producing either SRPS/ATD spectrum and/or CED have been identified in members of the ciliary intraflagellar transport-A (IFT-A) complex (Table 1): IFT144/*WDR19* [OMIM 608151], *IFT122/WDR140/WDR10* [OMIM 218330], IFT121/*WDR35* [OMIM 613610], and *IFT43* [OMIM 614099] that has thus far only been associated with CED [23–27]. The IFT-A complex [27, 28], together with the dynein 2 motor complex, mediates retrograde transport in cilia. There are three core components of the complex, IFT122, IFT140, and IFT144, and three satellite components, IFT43, IFT121, and IFT139 [29, 30], as shown in Fig. 3e. As characterized in *Chlamydomonas reinhardtii*, IFT-A complex stability appears to be dependent on an intact core, as loss of IFT122 resulted in decreased levels of the other core members, IFT140 and IFT144, undetectable levels of satellite members IFT121 and IFT139, and abnormal cytosolic localization of IFT43 [29]. By contrast, loss of satellite member IFT121 did not destabilize the IFT-A core complex, but did result in dissociation of IFT43 from the IFT-A core and its abnormal localization to the cytosol. Thus, incorporation of IFT43 into the IFT-A complex requires both an intact core and the presence of IFT121 [31].

**Table 1:** Ciliopathies caused by mutations in IFT-A genes

IFT-A gene/syndrome	ATD or Jeune syndrome	SRPS	CED	Other ciliopathies
*IFT122*			CED [26]	
*IFT140*	Jeune syndrome [12, 39] Mainzer-Saldino [12, 39]^a^			
*IFT144/WDR34*	Jeune syndrome [6, 40]		CED [6]	Senior-Loken Syndrome [6, 41, 42]
*IFT139/TTC21B*	Jeune syndrome [17]			Nephronophthisis 12 [17]
*IFT121/WDR35*		SRPS [13]	CED [25]	
*IFT43*		SRPS present report	CED [27]	

^a^Now considered the same disorder Jeune syndrome, SRPS, short rib polydactyly syndrome, CED, cranioectodermal dysplasia, ATD, asphyxiating thoracic dysplasia

In a cohort of skeletal ciliopathy patients, we identified *IFT43* mutations in two genetically independent cases of SRPS. The clinical and radiographic phenotype was consistent with a diagnosis of SRPS type II, but there were additional unusual abnormalities that included bent ribs and long bones, and under mineralization of the calvarium. A very similar radiographic phenotype was identified in two cases of SRPS resulting from *IFT121* (*WDR35*) mutations. Similar to the findings in *C. reinhardtii*, cells derived from patients with *IFT43* mutations showed that loss of functional IFT43 did not impact the stability of the IFT-A core complex; however, mutations in the IFT43 satellite complex partner IFT121 destabilized IFT43. The data thus identify additional genetic and phenotypic heterogeneity in SRPS and demonstrate that the hierarchy of IFT-A complex formation is evolutionarily conserved.

## Methods

### Collection of human samples, ethics, consent, and permissions

Under an approved University of California at Los Angeles human subjects protocol, informed consent was obtained from the participants and clinical information, radiographs, blood and tissue samples were obtained. All experimental protocols and methods were carried out in accordance with institutional guidelines and regulations and approved by the UCLA Institutional Review Board and Biosafety Committees.

### Exome analysis

DNA was isolated and submitted to the University of Washington Center for Mendelian Genomics for library preparation and exome sequencing in a cohort of skeletal ciliopathy cases. The samples were barcoded, captured using the NimbleGen SeqCap EZ Exome Library v2.0 probe library targeting 36.5 Mb of genome, and sequenced on the Illumina GAIIx platform with 50 bp bidirectional reads. Novoalign was used to align the sequencing data to the human reference genome (NCBI build 37) and the Genome Analysis Toolkit (GATK) was used for post-processing and variant calling according to GATK Best Practices recommendations. For each sample, at least 90% of targeted bases were covered by at least 10 independent reads. Variants were filtered against dbSNP137, NIEHS EGP exome samples (v.0.0.8), exomes from the NHLBI Exome Sequencing Project (ESP6500), 1000 genomes (release 3.20120430), and in-house exome samples. Mutations were further compared with known disease-causing mutations in HGMD (2012.2). Variants were annotated using VAX34, and mutation pathogenicity was predicted using the programs Polyphen35, Sift36, Condel37, CADD38, and MutationTaster. Potential disease-associated variants were identified under an autosomal recessive model, identifying either homozygosity for variants in one gene or compound heterozygosity for two variants in the same gene. Variants were then filtered based on whether the genes had known roles in cilia. The mutations reported in this work were confirmed by bidirectional Sanger sequencing of amplified DNA from the probands and the parents. Primer sequences were IFT43-ex1F GAGGATTTGCTCGACTTGGAC; IFT43-ex1R CTGCGCTGACTCCTGTTGAG; IFT43-ex78F CCCGGTTTTGTGAGAAAGAG; IFT43-ex78R GGAGGAGATGGCACAGAATAAG; WDR35-ex9F CTCTGTGAAGTGATTGGTGGT; WDR35-ex9R AGGCTTCCTTTCTGCTAGCT; WDR35-ex13F AGCCAAGAAGTTAAAGGAAGCA; WDR35-ex13R ATCCCCTGTCAAGCTAGCAA; WDR35-ex14F TGGTACAGCTTTCAGGAACG; WDR35-ex14R ACAGATGGGAAGGTGTGAGG; WDR35-ex16F ACTTTCCACCATGTTTCTCTAGA; WDR35-ex16R GATGCCTGCGCCTTCATAAC. Sequence trace files were aligned and analyzed using 4peaks.

### Cell culture

Low passage primary fibroblasts and amniocytes were cultured in DMEM with 10% FBS. Experiments were performed after starving cells in 0.5% serum for 48 h prior to cell collection.

### Western blot analysis

Each antibody was tested in at least three experimental replicates from independent cultures. For the analyses, there were two independent cases with either mutations in *IFT43* or *IFT121* mutations. In each western blot analysis, there were at least three experimental replicates with technical replicates. Cell lysates were collected from 80% confluent, starved cultures in RIPA buffer containing proteinase inhibitors (Sigma). Lysates were cleared by centrifugation and quantified (Pierce BCA protein assay kit) to attain equal loading. 40 μg of each lysate was loaded with Laemmli buffer and separated by 10% SDS-PAGE and transferred to PVDF membranes. Membranes were preincubated for 1 h and then incubated with antibody overnight in 5% milk in TBST. HRP secondary antibody and ECL-film exposure were used to detect proteins. Antibodies used as follows: IFT43 (Santa Cruz, N19, 1:100); WDR19/IFT144 (Abcam, 1:100); WDR35/IFT121 (Sigma HPA044147, 1:100); DYNC2L1 (Proteintech, 1:500) GLI3 (R&D Systems, 1:200); GAPDH (Cell Signaling, 1:1000). HRP secondary antibodies were Cell Signaling (1:1000, anti-mouse and -rabbit) and Santa Cruz (1:3000, anti-goat). FIJI was used to quantify proteins following Gel Analysis recommendations from ImageJ and Gassmann et al. [32] (http://rsb.info.nih.gov/ij/docs/menus/analyze.html#gels) and the Mann–Whitney test was used for statistical analysis using Prism software.

### Immunofluorescence and cilia measurements

Cells were cultured in 4-well chamber slides (LabTek). After serum starvation, cells were washed twice with PBS and fixed in PFA for 10 min and then permeabilized with 0.1% Triton-X-100 in PBS for 15 min at room temperature. Cells were then blocked in 10% goat serum for 1 h at room temperature. Primary antibodies were diluted in PBS containing 1% serum and incubated overnight at 4 °C. Antibodies used were as follows: ARL13B (Proteintech, 1:100); acetylated α-tubulin (Sigma T6793, 1:2000); pericentrin (Abcam, 1:2000). DAPI was used to stain the nuclei. Detection was performed with secondary Alexa Fluor 488/568 antibodies (Invitrogen, 1:1000). Images were captured on Zeiss Confocal 810. Images were collected with 1024 × 1024 pixel definition and Z-sections were taken at 0.5-µm step size. Max projections of the Z-stacks were used for primary cilium counting in ImageJ (NIH).

### Histology

Distal femurs from the probands were fixed in 10% formalin and then decalcified in Immunocal (Formic Acid) for three days, dehydrated, and embedded in paraffin. 10-µm sections were obtained and stained with picrosirius red for 30 min with hematoxylin as a counterstain.

## Results

### Clinical and radiographic findings

**Fig. 1:**
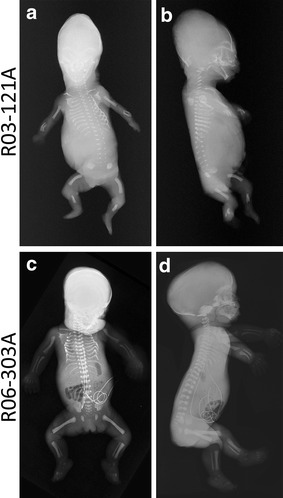
SRPS radiographic phenotype. **a**, **b** Radiographic findings in R03-121A. An 18-week gestational age fetus with short, narrow bent ribs, a bell-shaped chest, handlebar clavicles, curved humeri with short bent radii and ulnae, curved femurs, fibular aplasia and lack of calcification of the distal extremities. **c**, **d** Radiographic findings in R06-303A. 30-week gestational age fetus with dolichocephaly and a prominent occiput, a long narrow thorax with deformed ribs, micromelia, and poor mineralization of the distal limbs, mild platyspondyly, and abnormal ilia

We identified three cases from two families (International Skeletal Dysplasia Registry reference numbers R03-121A and R06-303A and R06-303E) with prenatal findings consistent with SRPS (Table 2). Affected individual R03-121A was from a non-consanguineous family of European ancestry, and prenatal ultrasound at 18 weeks gestational age showed a cystic hygroma, micromelia, a poorly calcified calvarium, and postaxial polydactyly of both hands and both feet. By history, similar findings were seen in this family’s first gestation but no postmortem information or material was available. The pregnancy was interrupted and radiographic and autopsy examination confirmed the SRPS diagnosis, but showed some unusual radiographic features that included short, irregularly bent ribs, short, campomelic long bones, hypoplastic and bent mesomelic bones, and lack of ossification of the calvarium, hands, and feet (Fig. 1a, b). Other organ system abnormalities included hydrocephalus, malrotation of the intestines, and polycystic kidneys (Table 2). The smooth appearance of the distal ends of the long bones and the presence of internal organ abnormalities were most consistent with a diagnosis of SRPS type II, but with the unusual radiographic findings noted above.

**Table 2:** Clinical findings in SRPS cases with IFT43 mutations

Case	R06-303A	R03-121	R03-342	R10-483
Clinical findings				
Diagnosis	SRP II	SRP II	SRPII	SRPII
Gestational age at delivery	30 weeks (R06-303A); 18.6 weeks (R06-303E)	18 weeks	22 weeks	23 weeks
Prenatal findings	Small chest, polydactyly, micromelia (both A and E)	Short thoracic circumference, polydactyly, cystic hygroma. Choroid plexus cysts, echogenic kidneys, micromelia	Cystic hygroma, echogenic kidneys and bowel, ascites, polydactyly, micromelia	Increased nuchal fold, hydrops, echogenic bowel, micromelia
Gene	*IFT43*	*IFT43*	*IFT121/WDR35*	*IFT121/WDR35*
Postnatal clinical and radiographic findings				
Cranium	Dolichocephaly	Poor mineralization of the calvarium	Scalp edema, low set ears	Scalp edema
Neuro	Brain with abnormal folding of the left hippocampus, neuroglial heterotopias in the roof of the temporal horn, and mildly dilated ventricles	Mild hydrocephalus	N/A	N/A
Eyes	Hypertelorism, bilateral epicanthal folds	No reported abnormalities	Hypertelorism	N/A
Mouth	Thin upper lip, attached to maxilla by mucosal fold, micrognathia	No reported abnormalities	Thin upper lip and micrognathia	N/A
Thorax	Small chest, abnormally bent ribs, mild platyspondyly	Narrow and barrel shaped chest, short, bent and decreased number of ribs (11), vertebrae flattened and abnormally wedged with round anterior ends	Narrow and barrel shaped chest, short and bent ribs, handlebar clavicles	Very short, variably bent ribs, handlebar clavicles
Gastrointestinal	Liver with ductal abnormalities, pancreas with stellate area of fibrosis in the tail	Malrotation of the intestines	N/A	N/A
Renal	Abnormal maturation of the kidneys with a poorly formed nephrogenic zone, thin cortex and medulla, and fibrosis	Polycystic kidneys	N/A	N/A
Upper extremities	Micromelia, reverse campomelia of humeri, curved radii, and ulnae	Micromelia, decreased mineralization with curved radii and ulnae	Micromelia with bowing of the radii and ulnae	Micromelia with bowing of the radii and ulnae
Pelvis	Abnormal ilia	Abnormal Ilia with decreased height, narrow sciatic notch, hypoplastic ischium	Abnormal ilia with abnormal absent sciatic notch and unformed acetabular roof	Flat acetabular roof, narrow sacrosciatic notch
Lower extremities	Micromelia, thin fibulae	Micromelia, angulated femur, hypoplastic tibae and fibulae	Micromelia	Micromelia, bending of the tibae and fibulae
Hands and feet	Postaxial polydactyly with brachydactyly, bilateral simian creases, bilateral partial syndactyly of the second and third toes	Preaxial polydactyly, brachydactyly and aphalangia in hands	Postaxial polydactyly in the hands and feet, and aphalangia in the hands	Poor mineralization of the hands and feet, no polydactyly

*N/A* not available

The second family, R06-303, also had two fetuses with SRPS. In the first pregnancy, with dizygotic twins, prenatal ultrasound revealed SRPS features in one twin. Delivery occurred at 30 weeks gestation and the affected newborn died the next day. Significant findings included short long bones with reverse campomelia of the humeri, bending of the bones of the mesomelic segments, a long narrow chest with bent ribs, postaxial polydactyly of all extremities (Fig. 1c, d) and brain, kidney, liver, and pancreas abnormalities (Additional file 1: Fig. S1) (Table 2). The second pregnancy was identified with similar findings and interrupted at 18 weeks gestational age. Similar to the affected individual studied in family R03-121, the findings were consistent with SRPS type II but also with the unusual finding of bending of the ribs and the mesomelic segments of the appendicular skeleton (Table 2). Recurrence of SRPS and historical consanguinity in family R06-303 (Additional file 2: Fig. S2) suggested a recessively inherited disorder due to homozygosity for a mutation, identical by descent.

### *IFT43* mutations identified in SRPS cases

**Fig. 2:**
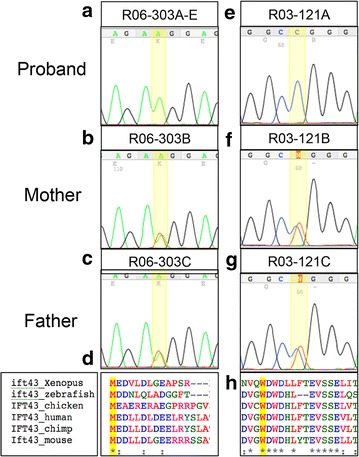
*IFT43* mutations in SRPS. **a**–**c** Chromatograms illustrating homozygosity for the p.Met1Lys *IFT43* mutation in proband R06-303A and heterozygosity in the parents. **e**–**g** Chromatograms illustrating homozygosity for the pTrp179Arg *IFT43* mutation in proband R06-303A and heterozygosity in the parents. **d** The mutation in R06-303A affects the *IFT43* start codon (highlighted in *yellow*). **h** The mutation in R03-121A alters a highly conserved tryptophan residue (highlighted in *yellow*)

Exome sequence analyses of affected individuals in both families identified putative causative mutations in *IFT43* [OMIM 61406], which encodes a component of the intraflagellar transport A (IFT-A) complex. Individuals R06-303A and E were homozygous for a start-loss variant, c.2T>A, predicted to result in the amino acid substitution, p.Met1Lys that would disrupt the initiation codon for IFT43 synthesis. Sanger sequence analysis confirmed the mutation, demonstrated that both affected siblings were homozygous for the mutation, and showed that both parents were carriers (Fig. 2a–c). The c.2T>A variant is novel, not found in dbSNP (https://www.ncbi.nlm.nih.gov/projects/SNP/), 1000 Genomes Project (www.internationalgenomes.org) and the Exome Aggregation Consortium (ExAC) (http://exac.broadinstitute.org) and was predicted to be damaging by SIFT and PolyPhen, with the MutationTaster prediction algorithm (http://www.mutationtaster.org) generating a probability of 0.999 by Bayes classifier.

Individual R03-121A was homozygous for the missense variant c.535T>C, predicted to result in the amino acid substitution p.Trp179Arg, and the parents were shown to be carriers (Fig. 2e–g). This variant was also novel and predicted to be damaging by SIFT and PolyPhen, with the MutationTaster prediction algorithm generating a probability of 0.999. Trp179 is a highly evolutionarily conserved residue among vertebrates (Fig. 2h) and the variant falls within a highly conserved region of IFT43.

### Effect of *IFT43* mutations on IFT-A complex

**Fig. 3:**
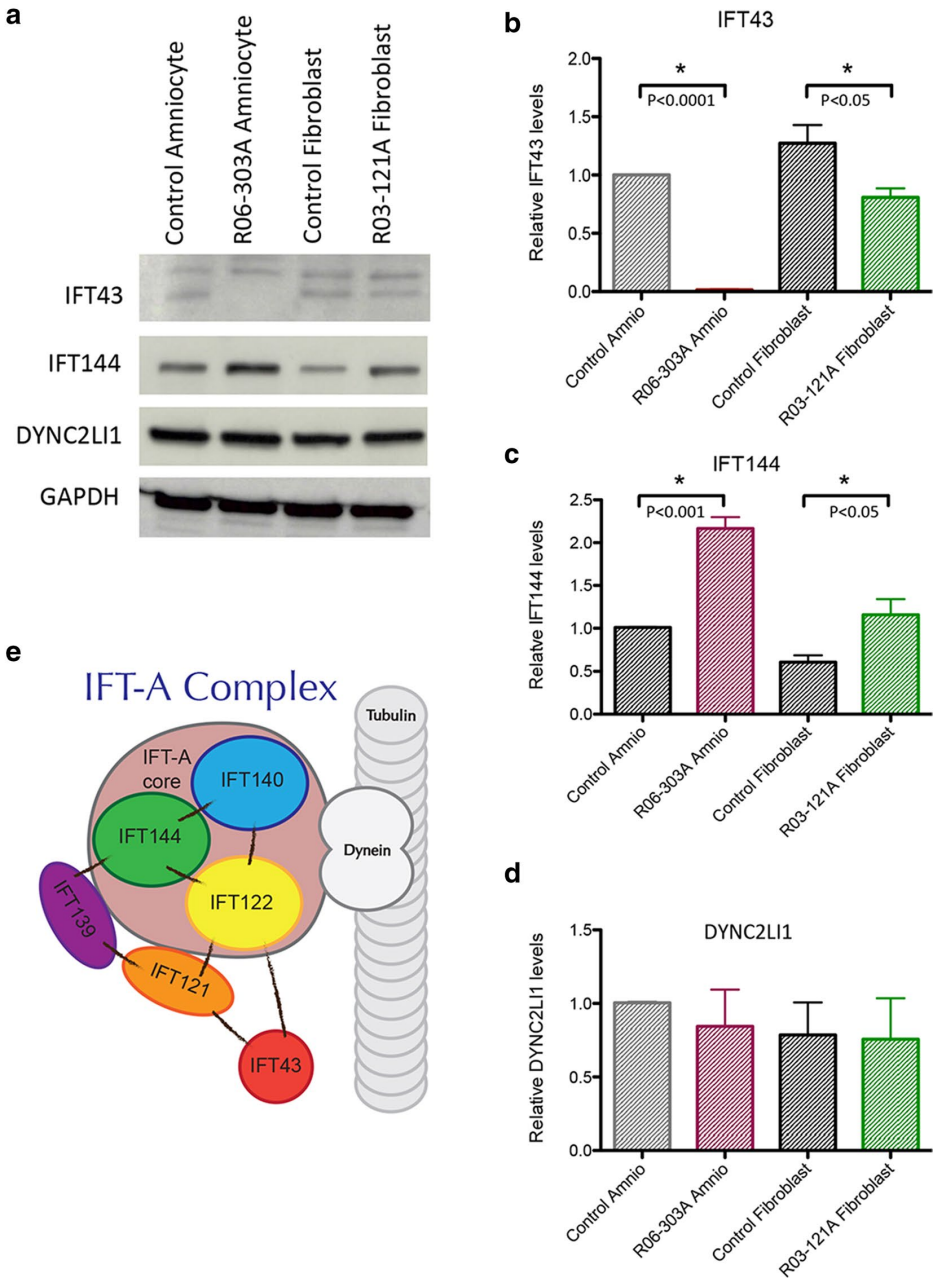
The IFT-A complex is altered in *IFT43* mutants. **a**, **b** The absence of IFT43 in R06-303A amniocytes relative to control and reduction of IFT43 protein in R03-121A fibroblasts compared with control. **a**, **c**, **d** Levels of WDR19/IFT144, and motor protein DYNC2LI1 in control and mutant cells. GADPH served as a loading control. *Bar graphs* show statistical analyses (*t* test) for the replicates (*n* = 3) of each studied protein. **e** Cartoon of the core and satellite IFT-A complex proteins. Lines connecting the proteins represent known interactions

Cultured fibroblasts and amniocytes from cases R03-121A and R06-303A, respectively, were used to assess the effect of the mutations on IFT43 protein levels and on other members of the IFT-A complex. By Western blot analyses (Fig. 3a), the IFT43 antibody generated a double band around the expected 43KDa in both cell lines; however, only the lower band showed altered levels in the *IFT43* mutant cells, suggesting that only the lower band was specific. R06-303A (p.Met1Lys) cells showed the absence of IFT43 while R03-121A (p.Trp179Arg) cells showed a statistically significantly reduced amount of the protein (Fig. 3a, b). cDNA levels were ascertained by RT-PCR in RNA derived from the mutant cell lines and showed levels similar to control cells (data not shown). This suggests that the *IFT43* mutations altered the synthesis and/or stability of the protein and not gene expression.

As IFT43 is a component of the IFT-A complex, we analyzed the IFT-A core complex member IFT144 (WDR19), to determine the effects of loss of IFT43 on stability of the complex (Fig. 3e). Western blots showed an increase in the protein level of the IFT-A core member IFT144 (Fig. 3a, c) in both cell lines. To determine if defects in IFT43 affected levels of retrograde transport complex motor proteins, we studied DYNC2LI1, one of the dynein proteins that forms the motor complex and moves the IFT-A complex along the microtubules. We found that DYNC2LI1 levels (Fig. 3a, d) were unaltered in the mutant cells, suggesting that defects in IFT43 do not affect the overall stability of the retrograde motor complex.

### Cilia formation is disrupted in IFT43 mutant cells

**Fig. 4:**
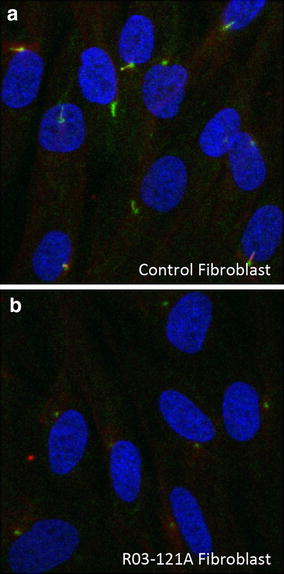
*IFT43* mutations induce ciliogenesis defects. **a**, **b** ARL13B (*green*) and Ac-Tub (*red*) staining of the cilia in control and R03-121A fibroblasts. Pericentrin (*green*) staining was used to mark the centrosome. Nuclei were stained in *blue*. R03-121A fibroblasts did not show cilia staining with ARL13B or Ac-Tub, only pericentrin centriole staining

Defects in retrograde transport components are known to affect the formation and/or architecture of primary cilia [3, 4, 15, 33]. To determine if mutations in *IFT43* altered ciliogenesis, we induced cilia formation in affected and control cells by culturing cells to near confluence followed by mild serum starvation. Under these conditions, neither control nor SRPS amniocytes formed cilia, so only the mutant fibroblasts derived from R03-121A (p.Trp179Arg) were analyzed in this experiment. As compared with controls, cilia were absent in *IFT43* mutant cells (Fig. 4a, b) indicating that the p.Trp179Arg *IFT43* mutation disrupted ciliogenesis.

### Cartilage growth plate abnormalities due to *IFT43* mutations

**Fig. 5:**
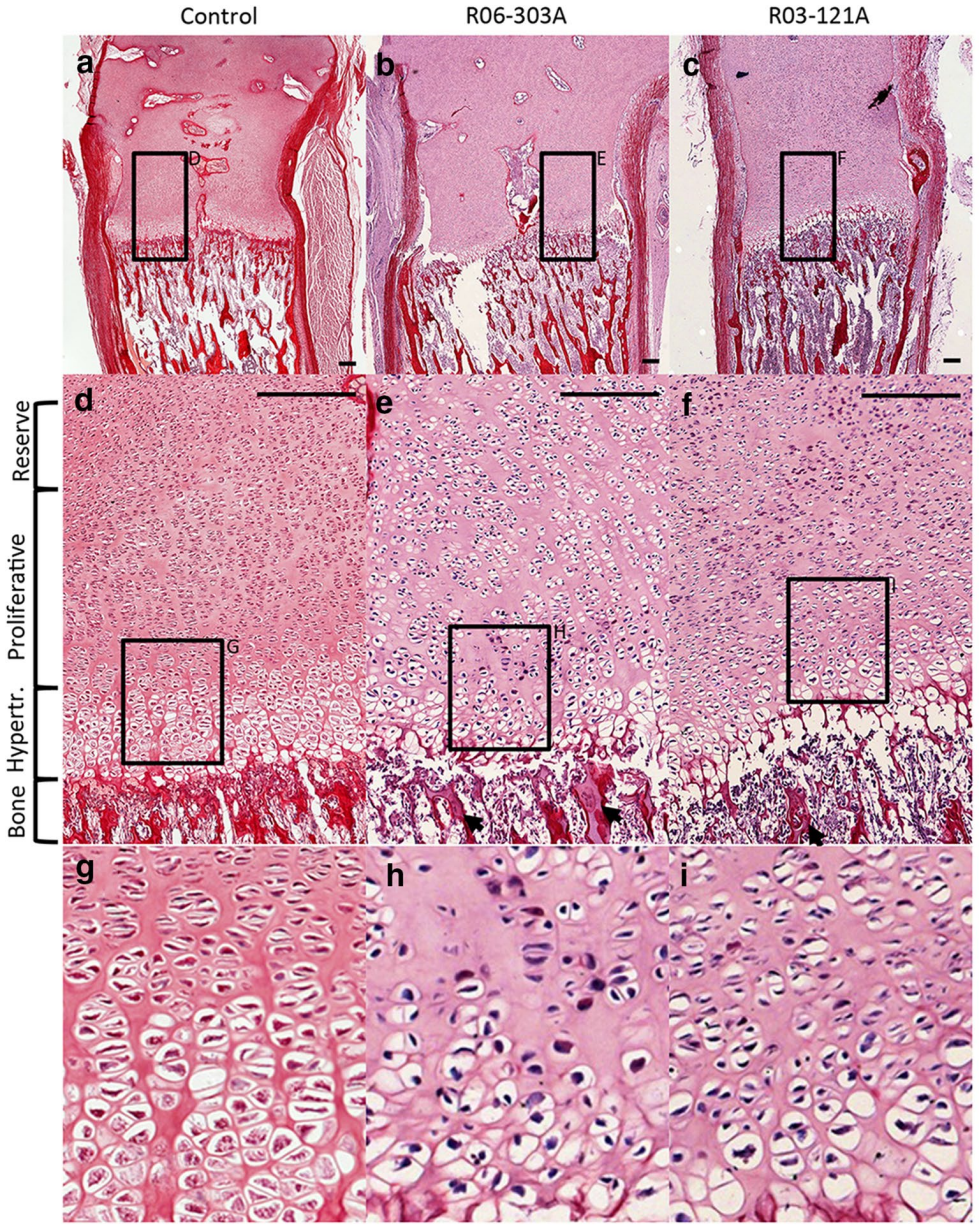
Growth plate defects in *IFT43* SRPS. **a**–**c** Picrosirius red and hematoxylin staining of cartilage growth plates in control and affected patients. **d**–**f** Expansion of boxed regions in **a**–**c**. Note the irregular column formation and lack of normal progressive enlargement of hypertrophic chondrocytes in both R06-303A and R03-121A. *Arrows* identify regions with retained cartilage in the primary spongiosum. **g**–**i** are magnifications in **d**–**f**, respectively showing polarity disruption in proliferating columns in patient samples. *Scale bars*, 50 μm. **g**–**i** pictures are also 50 μm wide

To determine the involvement of IFT43 in endochondral ossification, we stained histological sections of distal femur growth plates from the two cases with *IFT43* mutations with picrosirius red and hematoxylin (Fig. 5). In both cases, there was a significantly abnormal pattern of proliferation and differentiation of chondrocytes extending from the reserve through the hypertrophic zones. There was disruption of the polarity of the proliferating cells, with the columns of chondrons showing more than one plane of division (Fig. 5). The hypertrophic zones were irregular, reflecting the disrupted column formation, and there were decreased numbers of hypertrophic cells and variability in the orderly transition that results in the increased size characteristic of late hypertrophic chondrocytes (Fig. 5d–f). There was evidence of retained rests of cartilage within the primary spongiosum (arrows in Fig. 5e, f) suggesting that there may be altered cartilage to bone transition at the distal end of the growth plate.

### Mutations in *IFT121* affect the stability of IFT43 and lead to a similar SRPS phenotype

Based on studies done in *C. reinhardtii*, loss of IFT43 did not affect stability of IFT121 (WDR35) [29], but loss of IFT121 negatively influenced IFT43 levels. To test whether these findings were also seen in vertebrates, we interrogated our skeletal ciliopathy cohort for cases with *IFT121* mutations. Exome analysis identified two SRPS cases, R03-342 and R10-483, resulting from mutations in *IFT121* and from which cultured cells were available. R03-342A was heterozygous for the variants c.1433G>A; p.Arg478Lys and c.1579C>T; p.Gln527Ter. R10-483A was heterozygous for the variants c.932G>T; p.Trp311Leu and c.1501delC; p.Gln501LysfsTer10. Three of the variants were novel, not found in dbSNP (https://www.ncbi.nlm.nih.gov/projects/SNP/), 1000 Genomes Project (www.internationalgenomes.org), and the Exome Aggregation Consortium (ExAC) (http://exac.broadinstitute.org), and the fourth variant, p.Trp311Leu, had a very low allele frequency of 0.00002486 in ExAC. All variants were predicted to be damaging by SIFT and PolyPhen, with the MutationTaster prediction algorithm (http://www.mutationtaster.org) generating a probability of 0.999 for the missense variants and 1.0 for the frameshift mutation.

**Fig. 6:**
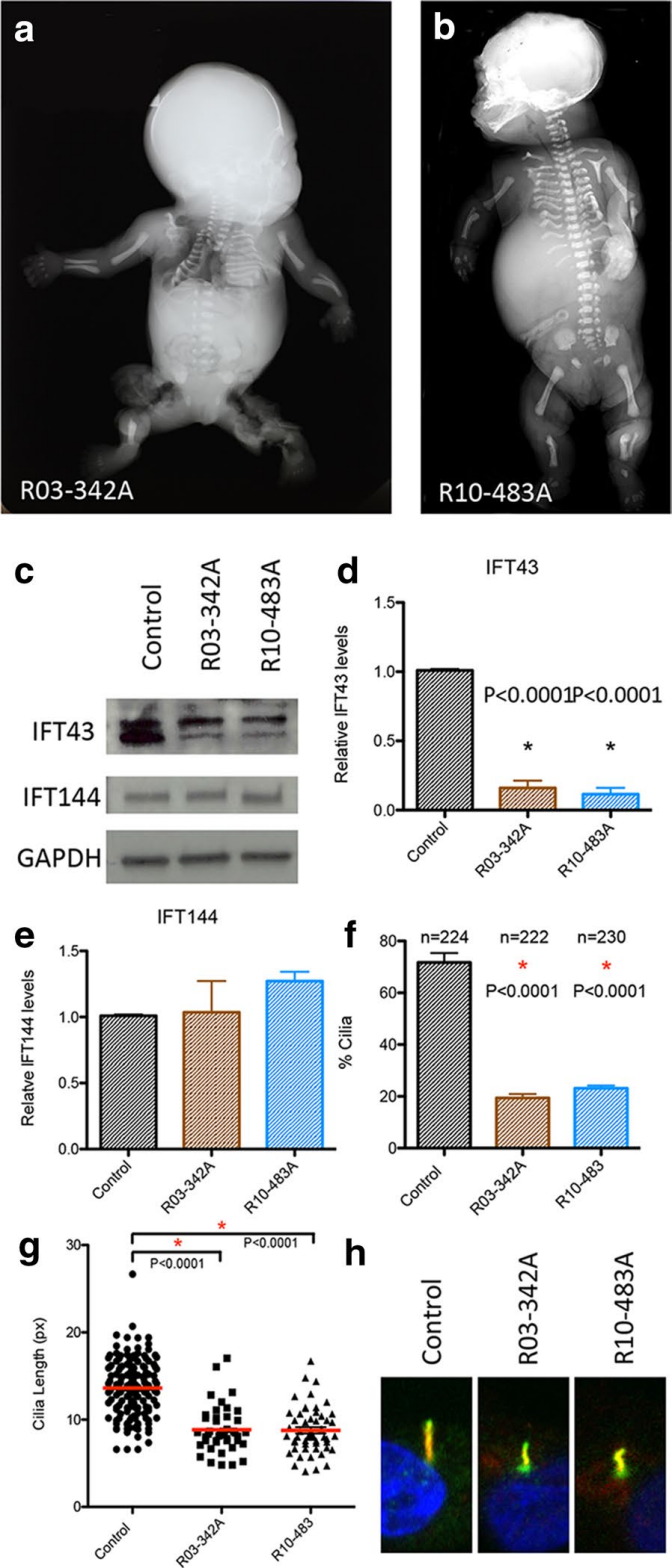
IFT121 mutations cause similar SRPS phenotype. **a** Radiographic findings in R03-342A with short, narrow bent ribs, a bell-shaped chest, curved humeri with short bent radii and ulnae. **b** R10-483 showed similar findings in thorax and extremities. **c**–**e** Patients (two independent cases) with mutations in IFT121 showed reduced IFT43 and similar levels of IFT144 (*N* = 3). **f**–**h** Both R03-342A and R10-483A showed a reduction in number of cilia (**f**) and length (**g**–**h**). ARL13B (*green*) and Ac-Tub (*red*) staining of the cilia in control and R03-121A fibroblasts. Pericentrin (*green*) staining was used to mark the centrosome. Nuclei in *blue*

The two cases had radiographic and clinical findings similar to the *IFT43* mutation cases (Fig. 6a, b; Table 2). Family R03-342 had three offspring with SRPS. The phenotype was characterized by short ribs, short limbs, bilateral polydactyly and bending of humeri, radii, and ulnae (Fig. 6a). Other significant findings included echogenic kidneys and bowel, scalp edema, and a cystic hygroma (Table 2). In the second family, R10-483, the affected female was diagnosed with SRPS during pregnancy and died one week after birth. Radiographic findings included micromelia, a small thorax with short and bent ribs, a pelvis with flat acetabular roofs, and bending of the distal long bones (Fig. 6b). Other abnormalities included skin edema and ascites (Table 2).

Analysis of cultured chondrocytes from the SRPS cases with *IFT121* mutations found decreased IFT43 levels (Fig. 6c, d) and normal levels of IFT-A core complex member IFT144 (Fig. 6c, e), indicating that while the *IFT121* mutations caused instability of the satellite component IFT43, they did not affect overall IFT-A core stability. Studies of the cilia in chondrocyte cell lines from both cases showed a reduction in the percentage of cilia present on the cells, indicating defective ciliogenesis (Fig. 6f). The average cilia lengths were reduced on the cells that had cilia and the cilia shapes were abnormal (Fig. 6g, h). These findings show that, similar to studies done in *C. reinhardtii*, loss of functional IFT121 does not affect the stability of the IFT-A core complex, but can influence the levels of satellite components. Interestingly, based on radiographs, the SRPS phenotypes in the *IFT43* and *IFT121* cases were very similar, including the unusual findings of campomelia and distinctive bending of the ribs. However, unlike the cells from the cases with the *IFT43* mutations, ciliogenesis in the *IFT121* cases was impaired but not absent.

## Discussion

The data presented here show that SRPS can result from recessively inherited mutations in *IFT43*, defining further locus heterogeneity for SRPS. Previously, homozygosity for an *IFT43* mutation was reported in a single family with cranioectodermal dysplasia (CED), a disorder with skeletal abnormalities similar to ATD but with the additional findings of craniosynostosis and ectodermal abnormalities [27]. Our data thus expand the spectrum of diseases caused by mutations in this gene to include a perinatal lethal skeletal ciliopathy phenotype. IFT43 is a satellite component of the IFT-A complex of intraflagellar retrograde transport. Mutations in the genes encoding other members of this complex, *IFT144* (*WDR19*), *IFT121* (*WDR35*), *IFT139 (TTC21B*), can produce CED [23–26], SRPS [13, 34], a distinctive form of EVC [35], and ATD [17] overlapping phenotypes that imply disruption of similar biological mechanisms when the IFT-A complex is defective.

With one published mutation in *IFT43* in CED [27] and our findings in two SRPS cases, it is unclear whether the clinical consequences of *IFT43* mutations, either CED or SRPS, depend on the specific nature of the identified mutations or on other genetic or stochastic factors. Interestingly, the CED mutation (c.1A>G; p.Met1Val) [27] and one of the SRPS mutations (c.2T>A; p.Met1Lys) both altered the initiating methionine codon, and both might be predicted to lead to loss of the protein. In the CED case, a construct containing the mutation was introduced into HEK293 cells and a stable protein of an apparent molecular weight consistent with use of a downstream methionine as the initiation codon was generated [27]. This result is concordant with in vitro translation data [36] showing that mutant start codons can still be recognized as a *bona fide* Kozak consensus sequences by the translational machinery and translation can be initiated at both the mutant non-AUG first codon and the next downstream methionine codon. This may be the explanation for the reported CED case that showed in vitro translation downstream of the initiating methionine mutation c.ATG>GTG. However, in our SRPS case, the initiating methionine mutation was c.ATG>AAG. AAG codon is the least efficient start codon that leads to use of a downstream methionine. Thus, this may explain why the c.ATG>AAG initiation codon mutation in our SRPS case led to loss of the IFT43 in cultured cells. Under this model, differences in translational output could explain why distinct mutations affecting the start codon might lead to different phenotypic outcomes. Furthermore, in the *IFT43* mutant CED case, the mutant fibroblasts were able to construct cilia and there was accumulation of IFT-A components at the cilia tips [27]. This is in contrast to the SRPS p.Trp179Arg fibroblasts, which showed a profound negative effect on ciliogenesis. Perhaps the extent of the effect on ciliogenesis also reflects the consequences of the different mutations, leading to the differences in phenotypic severity. Despite these inferences, we cannot rule out the possibility that the phenotypic differences between the CED and SRPS *IFT43* cases could be explained by factors beyond mutation specificity. For the SRPS cases studied here, in the exome analyses, there were no other variants in known cilia-related or ciliopathy disease-associated genes that would be predicted to influence the phenotype through increased genetic load [37].

Despite loss of IFT43, cultured cells from the cases synthesized an increased amount of IFT144, suggesting that the IFT-A core proteins are stable. Whether the increase in IFT144 reflects lack of proper recycling due to defective IFT or is a compensatory mechanism is unknown. Data derived from *C. reinhardtii* showed that loss of IFT121 reduced the level of IFT43 but that IFT43 did not influence the levels of its interacting partner protein IFT121 [29, 31], establishing a hierarchy among satellite components. Also, IFT43 gene is not conserved in all ciliated organisms, suggesting that IFT43 function might not be essential in some circumstances or at least ciliogenesis could adapt to the absence of IFT43. Our data in human cells support these findings, showing that mutant IFT121 dramatically destabilized IFT43. These data underscore the emerging importance of IFT121 as a central organizer for cilium structure and function. According to Fu et al. [38], IFT121 is critical for cilium assembly by selection of distinct cargoes for transport, functions in the development of Rab8 vesicles at the nascent cilium, is involved in protein exit from the cilium, and is important in centriolar satellite organization.

The recessively inherited mutations in *IFT43* and *IFT121* produced a very similar and distinctive SRPS phenotype, consistent with the findings from cells and model organisms that the encoded proteins directly interact. Through a study of a series of truncating mutations, the minimal IFT121 regions required for binding to other complex components were determined [38]. Based on these data, the SRPS missense mutations identified here are predicted to interfere with IFT121 binding to core complex member IFT122 and the centrosomal protein 290-KD (Cep290). While the *IFT121* missense mutations p.Arg478Lys and p.Trp311Leu (along with a null mutation) led to loss of IFT43, it is not clear whether this was due to disruption of the interaction between the altered region of IFT121 and the nearby region of interaction (residues 545–1181) with IFT43.

The phenotypic finding of campomelia of the long bones and the axial skeleton, poor mineralization, wavy, bent ribs has not been well described in the SRPS subtypes. Review of the two affected fetuses in a familial IFT121 SRPS cases [13, 34] showed the same phenotypic findings to our IFT43 and IFT121 cases presented here and were referred to as an unclassified form of SRPS. How these IFT-A mutations produce this distinct phenotype remains speculative. In part, the phenotype may result from the role of IFT-A in transport of cargos that include several G-protein coupled receptors including the serotonin receptor, the hedgehog component Smo, and possible other pathways components. Hedgehog signaling is essential for normal chondrocyte differentiation and coupling it to osteogenesis necessary for normal bone growth, mesenchymal cell differentiation, repair mechanisms, and limb bud patterning. Our findings show that functional IFT43 has a key role in cilia function necessary for normal skeletogenesis, and loss of IFT43 either directly due to mutation or as result of instability due to mutations in IFT121 has similar phenotypic skeletal consequences.

## Conclusions

The data presented here demonstrate that *IFT43* and *IFT121* mutations produce a similar and unique form of SRPS characterized by appendicular and axial campomelia and undermineralization of the skeleton. The data expand on the spectrum of phenotypes produced by *IFT43* mutations and identify a new locus for SRPS. Biochemical data show that *IFT121* mutations destabilize IFT43 but *IFT43* mutations did not destabilize the IFT-A core complex, suggesting evolutionary conservation of the hierarchy of complex formation from *C. reinhardtii* through humans. Despite the observation that diminished levels IFT43 did not appear to destabilize the IFT-A core complex, lack of adequate IFT43 levels disrupted ciliogenesis, suggesting that IFT43 may serve as a key component in initiating cilia formation.

## Additional files

**Additional file 1: Figure S1.** Clinical findings in R06-303A. Major findings in R06-303A were small thorax, poor ossification of the phalanges, polydactyly in both hands and feet (a, b), kidneys showing thin cortex and areas of fibrosis (c, e, f, g) and pancreas (d) showing cystic changes in the tail.

**Additional file 2: Figure S2.** Pedigree for families R03-121 and R06-303. Squares represent male family members, circles female family members, black symbols affected family members, double line consanguinity, dot carriers and triangles abortions.

## Abbreviations

SPRS: short rib polydactyly syndromes
IFT: intraflagellar transport
ATD: asphyxiating thoracic dystrophy
CED: cranioectodermal dysplasia
EVC: Ellis-van Creveld syndrome
WED: Weyers acrofacial dysostosis
Hh: hedgehog
