# Supplementary figures and images for: Mutations in IFT-A satellite core component genes IFT43 and IFT121 produce short rib polydactyly syndrome with distinctive campomelia
Source: Cilia. 2017 Apr 10;6:7. doi: 10.1186/s13630-017-0051-y (PMC5387211; doi:10.1186/s13630-017-0051-y)

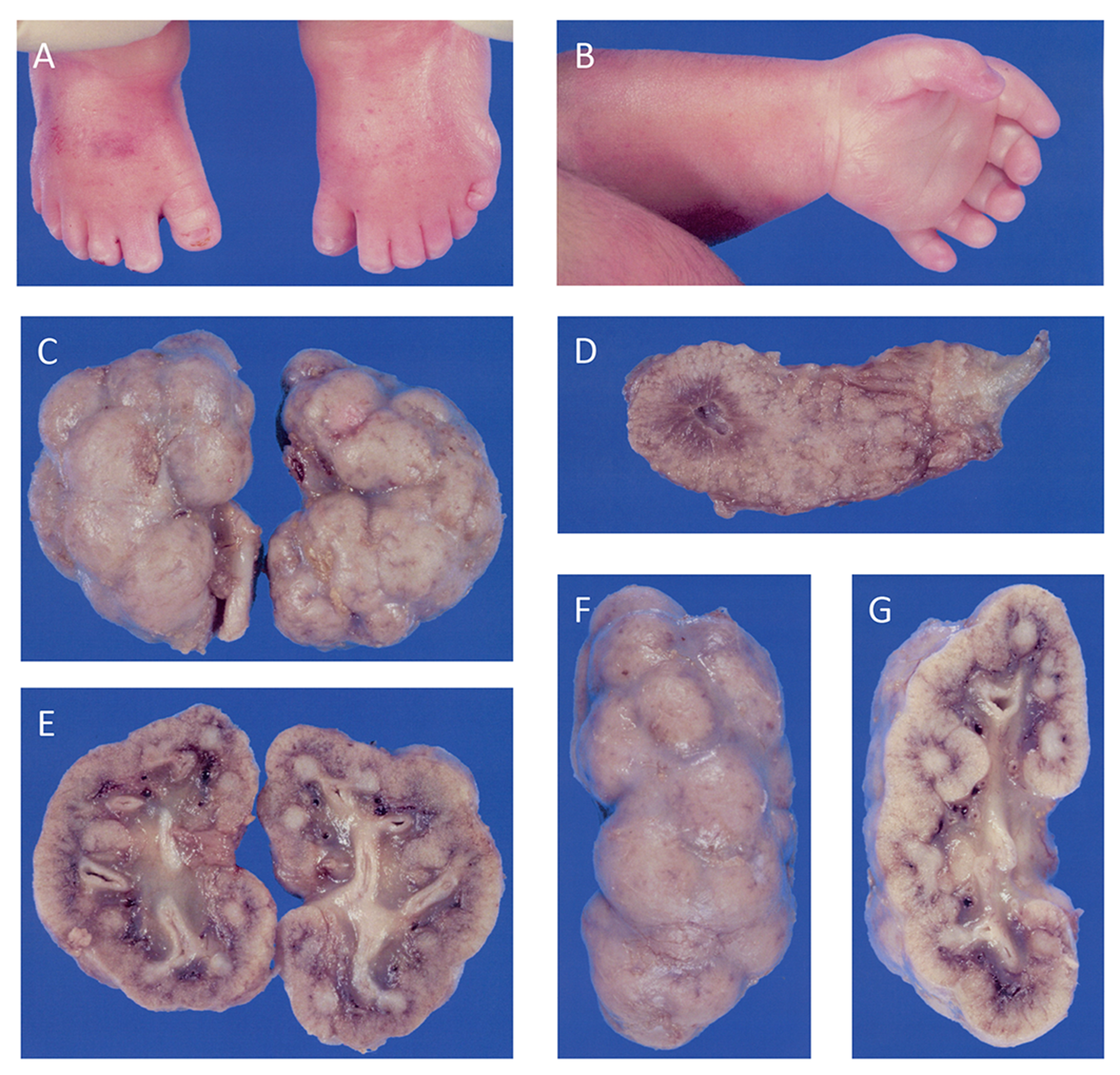

Supplement: Supplementary file 1 — Additional file 1: Figure S1. Clinical findings in R06-303A. Major findings in R06-303A were small thorax, poor ossification of the phalanges, polydactyly in both hands and feet (a, b), kidneys showing thin cortex and areas of fibrosis (c, e, f, g) and pancreas (d) showing cystic changes in the tail. [file 13630_2017_51_MOESM1_ESM.tif]

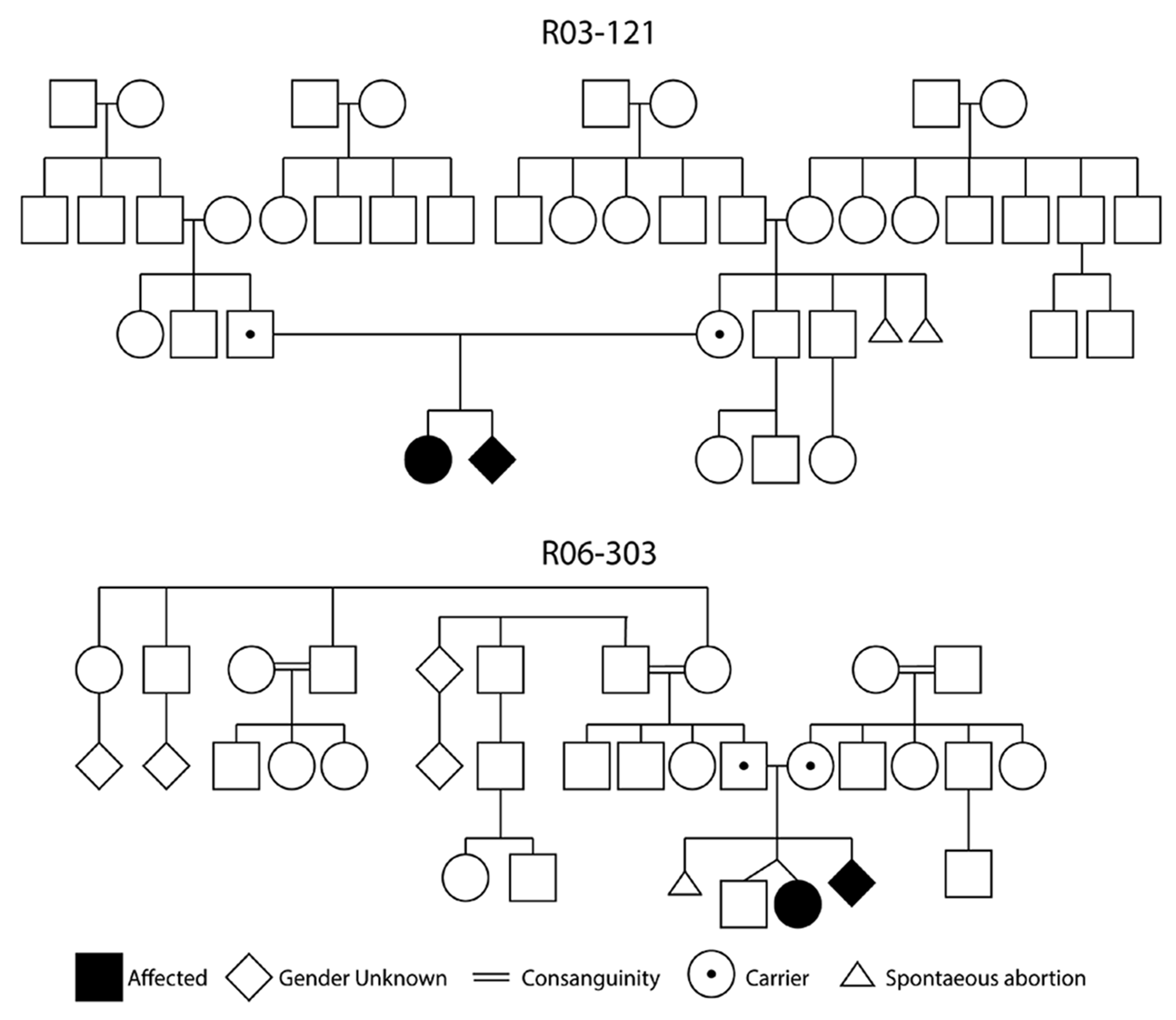

Supplement: Supplementary file 2 — Additional file 2: Figure S2. Pedigree for families R03-121 and R06-303. Squares represent male family members, circles female family members, black symbols affected family members, double line consanguinity, dot carriers and triangles abortions. [file 13630_2017_51_MOESM2_ESM.tif]
